# Structural Variety in Transition Metal Complexes of Tripodal Ligands Containing Mixed Quinolyl and Pyridyl Donors

**DOI:** 10.1002/open.202400304

**Published:** 2024-10-23

**Authors:** Bronte Carr, Timothy D. Christopher, Tilo Söhnel, Lawrence R. Gahan, Cassandra L. Fleming, Allan G. Blackman

**Affiliations:** ^1^ Department of Chemistry Auckland University of Technology Private Bag 92006 Auckland 1142 New Zealand; ^2^ School of Chemistry The University of Sydney Chemistry Building, Eastern Ave Camperdown NSW 2050 Australia; ^3^ School of Chemical Sciences University of Auckland Private Bag 92019 Auckland 1142 New Zealand; ^4^ MacDiarmid Institute for Advanced Materials and Nanotechnology Victoria University of Wellington PO Box 600 Wellington 6140 New Zealand; ^5^ School of Chemistry and Molecular Biosciences The University of Queensland Brisbane, Queensland 4072 Australia

**Keywords:** Tripodal, Quinoline, X-ray structure, Dimer, Calculations

## Abstract

The syntheses of the tripodal tetraamine ligands 2‐(pyridin‐2‐yl)‐N,N‐bis(quinolin‐2‐ylmethyl)ethan‐1‐amine (DQPEA), N‐(pyridin‐2‐ylmethyl)‐2‐(quinolin‐2‐yl)‐N‐(2‐(quinolin‐2‐yl)ethyl)ethan‐1‐amine (DQEPMA**)**, 2‐(pyridin‐2‐yl)‐N,N‐bis(2‐(quinolin‐2‐yl)ethyl)ethan‐1‐amine (DQEPEA), N,N‐bis(pyridin‐2‐ylmethyl)‐2‐(quinolin‐2‐yl)ethan‐1‐amine (QEDPMA), and 2‐(pyridin‐2‐yl)‐N‐(2‐(pyridin‐2‐yl)ethyl)‐N‐(2‐(quinolin‐2‐yl)ethyl)ethan‐1‐amine (QEDPEA) containing mixed quinolyl and pyridyl moieties are reported, with 2‐vinylquinoline being used to attach quinolylethyl arms to the aliphatic N atom. X‐ray crystal structures of [(Mn(DQPEA))_2_O_2_](ClO_4_)_2_ ⋅ (CH_3_CN)_2_, [Cu(DQPEA)NCCH_3_](ClO_4_)_2_, [Zn(DQPEA)NCCH_3_](ClO_4_)_2_, [Pd(DQEPEA)Cl]Cl ⋅ 11H_2_O are detailed, with four, five, and six‐coordination observed. In addition, the dimeric complex [(DPEA)Co(μ‐OH)_3_Co(DPEA)](ClO_4_)_3_ ⋅ 0.5H_2_O ⋅ MeCN containing the tridentate DPEA ligand formed by N‐dealkylation of QEDPEA is reported. Calculations suggest that the very short Co…Co distance of 2.5946(6) Å in this complex is unlikely to be due to a Co−Co bond.

## Graphical abstract

New tripodal tetraamine ligands containing mixed quinolyl and pyridyl donors have been prepared and their coordination chemistry studied. 2‐vinylquinoline has proven useful for the introduction of the hitherto unknown 2‐quinolylethyl unit. Crystal structures of Mn_2_(III,III), Co_2_(III,III), Cu(II), Zn(II) and Pd(II) complexes of these ligands are reported.

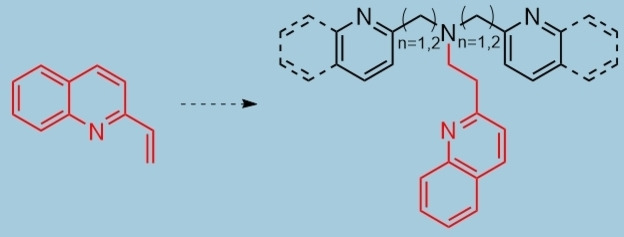

## Introduction

**Figure 1:**
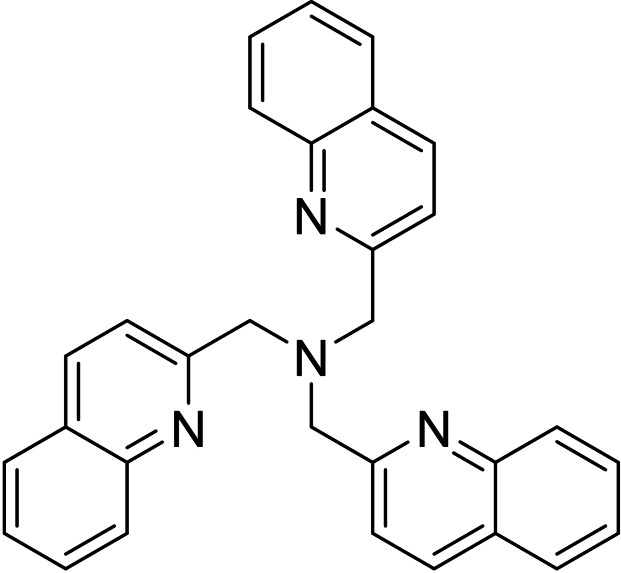
Structure of the TMQA ligand.

The synthesis and coordination chemistry of tripodal tetradentate ligands containing three pyridyl units has been a subject of increasing interest since the initial report of the ligand tris(2‐pyridylmethyl)amine (alternately abbreviated as both tpa and tmpa) in 1967.^[1]^ A significant number of such ligands, and complexes thereof, have since been prepared, with over 1000 complexes containing the unsubstituted tpa ligand alone reported in the CSD.[^2^, ^3^, ^4^] The use of substituted pyridines and alteration of the methylene chain lengths have resulted in the synthesis of many other tripodal tripyridyl ligands, and studies of metal complexes of these have shown that relatively small changes in the tripodal framework can have significant effects on structure, physical properties, and reactivity.[^2^, ^3^, ^5^] In contrast, the synthesis and coordination chemistry of quinolyl‐containing tripodal tetradentate ligands has been much less studied; for example, there are only 18 structurally characterised complexes of the quinoline analogue of tpa, tris(2‐quinolylmethyl)amine (TMQA, Figure 1), first prepared in 1994,^[6]^ and 4 other substituted analogues[^7^, ^8^, ^9^] constitute the only known tripodal tetradentate ligands containing three quinoline units.^[10]^ This is surprising, given the current interest in utilising quinoline‐containing ligands as fluorescent sensors, particularly for Zn^2+^.[^11^, ^12^, ^13^]

More numerous, yet still relatively rare, are tripodal ligands containing both quinolyl and pyridyl moieties, and a small number of these have been prepared and their coordination chemistry studied.^[10]^ However, none of these contain the 2‐quinolylethyl moiety, and given that there is, remarkably, only a single example of a 2‐aminoethylquinoline fragment reported in the CSD,^[14]^ we were interested in preparing ligands containing this unit. In this paper, we report the synthesis of four new tripodal ligands containing the unprecedented 2‐quinolylethyl moiety, and an investigation of their coordination chemistry. We also report the first synthesis of the DQPEA ligand, and structurally characterised Cu(II) and Zn(II) complexes of this.

## Experimental

### General Remarks

^1^H and ^13^C NMR spectra were recorded on a Bruker Avance 400 MHz spectrometer. High‐Resolution mass spectra were recorded on a Bruker micrOTOF−Q II mass spectrometer in positive electrospray ionization (ESI) mode. X‐ray data were recorded on a Rigaku Oxford Diffraction XtaLAB‐Synergy‐S single crystal diffractometer with a PILATUS 200 K hybrid pixel array detector using Cu Kα radiation (λ=1.54184 Å). The data were processed with the SHELX^[15]^ and Olex2[^16^, ^17^] software packages. All non‐hydrogen atoms were refined anisotropically. Hydrogen atoms were inserted at calculated positions and refined with a riding model or without restrictions. Deposition Numbers 2365256 ([Pd(DQEPEA)Cl]Cl ⋅ 11H_2_O), 2365257 ([(Mn(DQPEA))_2_O_2_](ClO_4_)_2_ ⋅ (CH_3_CN)_2_), 2365258 ([Zn(DQPEA)NCCH_3_](ClO_4_)_2_), 2365259 ([(DPEA)Co(μ‐OH)_3_Co(DPEA)](ClO_4_)_3_ ⋅ 0.5H_2_O ⋅ MeCN) and 2365260 ([Cu(DQPEA)NCCH_3_](ClO_4_)_2_) contain the supplementary crystallographic data for this paper. These data are provided free of charge by the joint Cambridge Crystallographic Data Centre and Fachinformationszentrum Karlsruhe Access Structures service. The density functional theory (DFT) calculations for the [(DPEA)Co(μ‐OH)_3_Co(DPEA)]^3+^ cation were performed with Gaussian 16,^[18]^ and geometry optimizations employed the M06 functionals^[19]^ and a mixed basis set consisting of LANL2DZ on the metal and 6–311G(d,p) on other atoms. The ultrafine integration grid of Gaussian 16 was used. All the calculations were performed in SMD implicit water,^[20]^ and the starting coordinates were obtained from the crystal structure.

### Syntheses

1‐(pyridin‐2‐yl)‐*N*‐[(pyridin‐2‐yl)methyl]methanamine^[21]^ and 2‐(pyridin‐2‐yl)‐*N*‐[2‐pyridin‐2‐yl)ethyl]ethan‐1‐amine^[22]^ were prepared according to literature methods.

### Synthesis of 2‐(bromomethyl)quinoline

This was prepared by modification of the method of Zhao at al.^[23]^ 1,1’‐azobis(cyclohexanecarbonitrile) (0.105 g, 0.429 mmol) was added to a solution of 2‐methylquinoline (3.07 g, 21.4 mmol) and NBS (4.20 g, 23.6 mmol) in benzene (100 mL) and the reaction was heated at reflux for 18 h. The resulting solution was washed with Na_2_CO_3_ (sat. aq. solution, 2×100 mL) and brine (sat. aq. solution, 2×100 mL), then dried over MgSO_4_, filtered, and the solvent was removed (rotavap). Purification by silica gel column chromatography (EtOAc/Hexane 1 : 1) afforded the title compound (2.15 g, 45 %); ^1^H NMR (400 MHz, CDCl_3_) δ 8.18 (dd, J=8.5, 0.8 Hz, 1H), 8.07 (dq, J=8.5, 0.9 Hz, 1H), 7.82 (dd, J=8.1, 1.5 Hz, 1H), 7.73 (ddd, J=8.5, 6.9, 1.5 Hz, 1H), 7.62–7.52 (m, 2H), 4.72 (s, 2H). ^13^C NMR (101 MHz, CDCl_3_) δ 156.75, 147.38, 137.14, 129.80, 129.12, 127.36, 127.18, 126.88, 121.01, 77.16, 76.84, 76.53, 34.22; LRMS (ESI, m/z): calculated for C_10_H_9_NBr^+^ [M+H]^+^ *m/*z=221.99; found *m/*z=221.9. Data are consistent with those previously reported.^[24]^

### Synthesis of 2‐vinylquinoline

This was prepared by modification of the method of Xiao et al.^[25]^ To a solution of 2‐methylquinoline (6.01 g, 41.9 mmol) in 1,4‐dioxane (100 mL), was added diethylamine hydrochloride (5.89 g, 54.4 mmol), formaldehyde solution (37 %, 6.5 mL, 54.5 mmol) and Et_3_N (0.88 mL, 6.3 mmol). After stirring at reflux for 1 h, the reaction mixture was cooled to room temperature and the solvent was removed (rotavap). The crude residue was dissolved in CH_2_Cl_2_ (100 mL) and washed with H_2_O (100 mL). The organic phase was washed with KOH(aq) (10 %, 2×25 mL), H_2_O (100 mL), dried over MgSO_4_, and reduced to dryness (rotavap) following filtration. Purification by silica gel column chromatography (4 % MeOH in CH_2_Cl_2_) afforded the title compound as a yellow/brown oil (6.11 g, 94 %); ^1^H NMR (400 MHz, CDCl_3_) δ 8.11 (d, *J=*8.6 Hz, 1H), 8.07 (dd, *J=*8.5, 0.8 Hz, 1H), 7.78 (dd, *J=*8.2, 1.5 Hz, 1H), 7.69 (ddd, *J=*8.4, 6.9, 1.5 Hz, 1H), 7.60 (d, *J=*8.6 Hz, 1H), 7.50 (ddd, *J=*8.1, 6.9, 1.2 Hz, 1H), 7.04 (dd, *J=*17.7, 10.9 Hz, 1H), 6.28 (dd, *J=*17.7, 0.9 Hz, 1H), 5.67 (dd, *J=*10.9, 0.9 Hz, 1H). ^13^C NMR (101 MHz, CDCl_3_) δ 156.23, 148.16, 138.13, 136.50, 129.79, 129.52, 127.62, 127.60, 126.46, 119.99, 118.51. LRMS (ESI, m/z): calculated for C_11_H_10_N^+^ [M+H]^+^ *m/z=*156.08; found *m/z=*156.1.

### Synthesis of 2‐(pyridin‐2‐yl)‐N,N‐bis(quinolin‐2‐ylmethyl)ethan‐1‐amine (DQPEA)

To a solution of 2‐(bromomethyl)quinoline (1.13 g, 5.11 mmol) in THF (20 mL), was added (*i‐*Pr)_2_NEt (1.7 mL, 10.2 mmol) followed by 2‐(pyridin‐2‐yl)ethan‐1‐amine (0.312 g, 2.56 mmol). After stirring at room temperature for 3 days, the reaction mixture was filtered through Celite and the solvent was removed (rotavap). The resulting crude residue was dissolved in CH_2_Cl_2_ (100 mL), and washed with NaOH(aq) (1 M, 50 mL) and H_2_O (100 mL), dried over MgSO_4_ and taken to dryness (rotavap). Purification by column chromatography on alumina (10 % MeOH in CH_2_Cl_2_+1 % NH_4_OH) afforded the title compound as a yellow/brown solid (0.951 g, 46 %); ^1^H NMR (400 MHz, CDCl_3_) δ 8.46–8.39 (m, 1H), 8.04 (d, *J*=8.4 Hz, 4H), 7.76 (dd, *J*=8.2, 1.6 Hz, 2H), 7.67 (ddd, *J*=8.4, 6.8, 1.5 Hz, 2H), 7.53 (dd, *J*=8.5, 1.5 Hz, 2H), 7.51–7.45 (m, 3H), 7.08 (ddd, *J*=7.6, 4.9, 1.2 Hz, 1H), 7.05 (dt, *J*=7.8, 1.1 Hz, 1H), 4.08 (s, *J*=1.4 Hz, 4H), 3.08 (s, *J*=2.2 Hz, 4H). ^13^C NMR (101 MHz, CDCl_3_) δ 160.52, 160.45, 149.06, 147.55, 136.40, 136.34, 129.46, 129.03, 127.56, 127.45, 126.22, 123.48, 121.23, 121.14, 61.16, 54.78, 35.90. HRMS (ESI, m/z): calculated for C_27_H_25_N_4_^+^ [M + H]^+^ *m/*z=405.2070; found 405.2076.

### Synthesis of N‐(pyridin‐2‐ylmethyl)‐2‐(quinolin‐2‐yl)‐N‐(2‐(quinolin‐2‐yl)ethyl)ethan‐1‐amine (DQEPMA)

To a stirred solution of 1‐(pyridin‐2‐yl)methanamine (0.802 g, 7.41 mmol) in 1,4‐dioxane (100 mL), was added 2‐vinylquinoline (2.30 g, 14.8 mmol) and Et_3_N (2.3 mL, 16.3 mmol). After stirring at reflux for 7 days, the reaction mixture was cooled to room temperature and KOH(aq) (10 %, 50 mL) was added. The mixture was extracted with CH_2_Cl_2_ (3×50 mL) and the combined organic fractions were washed with H_2_O (100 mL), dried over MgSO_4_ and concentrated (rotavap) following filtration. Purification by column chromatography on alumina (4 % MeOH in CH_2_Cl_2_) afforded the title compound as a brown oil (1.52 g, 49 %); ^1^H NMR (400 MHz, CDCl_3_) δ 8.44 (ddd, *J=*4.9, 1.8, 0.9 Hz, 1H), 7.98 (dq, *J=*8.5, 0.8 Hz, 2H), 7.83 (dd, *J=*8.4, 0.8 Hz, 2H), 7.73 (dd, *J=*8.2, 1.4 Hz, 2H), 7.66 (ddd, *J=*8.5, 6.9, 1.5 Hz, 2H), 7.47 (ddd, *J=*8.1, 6.9, 1.2 Hz, 2H), 7.18 (td, *J=*7.6, 1.8 Hz, 1H), 7.08 (d, *J=*8.4 Hz, 2H), 6.99 (ddd, *J=*7.5, 4.9, 1.2 Hz, 1H), 6.95 (dt, *J=*7.8, 1.1 Hz, 1H), 3.91 (s, 2H), 3.21–3.06 (m, 8H). ^13^C NMR (101 MHz, CDCl_3_) δ 161.33, 160.14, 148.74, 147.97, 136.19, 135.94, 129.39, 128.90, 127.56, 126.85, 125.81, 122.98, 122.06, 121.81, 60.37, 54.19, 36.89. HRMS (ESI, m/z): calculated for C_28_H_27_N_4_^+^ [M + H]^+^ *m/z=*419.22302; *m/z=*found 419.2228.

### Synthesis of 2‐(pyridin‐2‐yl)‐N,N‐bis(2‐(quinolin‐2‐yl)ethyl)ethan‐1‐amine (DQEPEA)

To a stirred solution of 2‐(pyridin‐2‐yl)ethan‐1‐amine (1.40 g, 11.4 mmol) in 1,4‐dioxane (100 mL), was added 2‐vinylquinoline (2.13 g, 13.7 mmol) and Et_3_N (3.2 mL, 22.8 mmol). After stirring at reflux for 7 days, the reaction mixture was cooled to room temperature and the solvent was removed (rotavap). The crude residue was dissolved in CH_2_Cl_2_ (100 mL) and washed with H_2_O (150 mL). The organic phase was washed with KOH(aq) (10 %, 2×20 mL), H_2_O (100 mL), dried over MgSO_4_ and concentrated (rotavap). Purification by column chromatography on alumina (4 % MeOH in CH_2_Cl_2_) afforded the title compound (2.06 g, 42 %) as a brown oil; ^1^H NMR (400 MHz, CDCl_3_) δ 8.49 (ddd, *J=*4.9, 1.9, 0.9 Hz, 1H), 8.03 (dq, *J=*8.5, 0.8 Hz, 2H), 7.85 (dd, *J=*8.4, 0.8 Hz, 2H), 7.73 (dd, *J=*7.9, 1.4 Hz, 2H), 7.67 (ddd, *J=*8.4, 6.9, 1.5 Hz, 2H), 7.48 (ddd, *J=*8.1, 6.9, 1.2 Hz, 2H), 7.35 (td, *J=*7.7, 1.9 Hz, 1H), 7.07 (d, *J=*8.4 Hz, 2H), 7.03 (ddd, *J=*7.6, 4.9, 1.2 Hz, 1H), 6.90 (dt, *J=*7.8, 1.1 Hz, 1H), 3.11 (d, *J=*1.7 Hz, 8H), 3.07–2.99 (m, 2H), 2.95–2.87 (m, 2H). ^13^C NMR (101 MHz, CDCl_3_) δ 161.55, 160.77, 149.23, 148.01, 136.16, 135.98, 129.42, 128.92, 127.59, 126.89, 125.81, 123.59, 122.12, 121.11, 54.00, 53.92, 36.99, 36.19. (HRMS) (ESI, m/z): calculated for C_29_H_29_N_4_^+^ [M + H]^+^ *m/*z=433.23867; *m/z=*found 433.2384.

### Synthesis of N,N‐bis(pyridin‐2‐ylmethyl)‐2‐(quinolin‐2‐yl)ethan‐1‐amine (QEDPMA)

To a stirred solution of 1‐(pyridin‐2‐yl)‐*N*‐[(pyridin‐2‐yl)methyl]methanamine (0.501 g, 2.51 mmol) in 1,4‐dioxane (50 mL), was added 2‐vinylquinoline (0.426 g, 2.74 mmol) and Et_3_N (0.38 mL, 2.25 mmol). After stirring at reflux for 6 days, the reaction mixture was cooled to room temperature and the solvent was removed under reduced pressure. The resulting crude residue was dissolved in CH_2_Cl_2_ (50 mL) and washed with H_2_O (100 mL). The organic phase was washed with KOH(aq) (10 %, 2×10 mL), H_2_O (100 mL), dried over MgSO_4_, and concentrated (rotavap) following filtration. Purification by alumina column chromatography (4 % MeOH in CH_2_Cl_2_) afforded the title compound as a brown oil (0.556 g, 63 %); ^1^H NMR (400 MHz, CDCl_3_) δ 8.48 (ddt, *J=*4.1, 2.2, 1.1 Hz, 2H), 8.02 (dd, *J=*8.5, 0.8 Hz, 1H), 7.97 (dq, *J=*8.5, 0.7 Hz, 1H), 7.78 (dd, *J=*8.0, 1.4 Hz, 1H), 7.68 (ddd, *J=*8.4, 6.9, 1.5 Hz, 1H), 7.50 (ddd, *J=*8.1, 6.9, 1.2 Hz, 1H), 7.44 (td, *J=*7.7, 1.9 Hz, 2H), 7.27 (d, *J=*6.6 Hz, 2H), 7.21 (d, *J=*8.4 Hz, 1H), 7.08 (ddd, *J=*7.4, 4.9, 1.2 Hz, 2H), 3.91 (s, 4H), 3.23 (td, *J=*6.8, 0.9 Hz, 2H), 3.13–3.03 (m, 2H). ^13^C NMR (101 MHz, CDCl_3_) δ 161.34, 159.88, 149.01, 148.02, 136.41, 136.09, 129.45, 129.27, 128.99, 127.62, 125.91, 122.99, 122.10, 122.00, 60.42, 54.40, 36.97. LRMS (ESI, *m/z*): calculated for C_23_H_23_N_4_^+^ [M + H]^+^ *m/z=*355.19; found *m/z=*355.1.

### Synthesis of 2‐(pyridin‐2‐yl)‐N‐(2‐(pyridin‐2‐yl)ethyl)‐N‐(2‐(quinolin‐2‐yl)ethyl)ethan‐1‐amine (QEDPEA)

To a stirred solution of 2‐(pyridin‐2‐yl)‐*N*‐[2‐pyridin‐2‐yl)ethyl]ethan‐1‐amine (4.04 g, 17.7 mmol) in 1,4‐dioxane (150 mL), was added 2‐vinylquinoline (3.31 g, 21.3 mmol) and Et_3_N (2.7 mL, 19.5 mmol). After stirring at reflux for 6 days, the reaction mixture was cooled to room temperature and the solvent was removed (rotavap). The crude residue was dissolved in CH_2_Cl_2_ (150 mL) and washed with H_2_O (150 mL). The organic phase was washed with KOH(aq) (10 %, 2×35 mL), H_2_O (150 mL), dried over MgSO_4_, and concentrated (rotavap) following filtration. Purification by column chromatography on alumina (4 % MeOH in CH_2_Cl_2_) afforded the title compound as a brown oil (2.55 g, 38 %); ^1^H NMR (400 MHz, CDCl_3_) δ 8.48 (ddd, *J=*4.9, 1.9, 0.9 Hz, 2H), 8.07–8.00 (m, 1H), 7.96 (dd, *J=*8.5, 0.8 Hz, 1H), 7.75 (dd, *J=*8.2, 1.5 Hz, 1H), 7.66 (ddd, *J=*8.5, 6.9, 1.5 Hz, 1H), 7.50–7.44 (m, 2H), 7.42 (dd, *J=*7.6, 1.9 Hz, 1H), 7.14 (d, *J=*8.4 Hz, 1H), 7.05 (ddd, *J=*7.6, 4.9, 1.2 Hz, 2H), 6.95 (dt, *J=*7.8, 1.1 Hz, 2H), 3.10–3.07 (m, 4H), 3.05–2.96 (m, 4H), 2.91 (m, 4H). ^13^C NMR (101 MHz, CDCl_3_) δ 161.40, 160.64, 160.62, 149.18, 147.94, 136.29, 136.27, 136.15, 136.13, 129.45, 128.84, 127.56, 126.88, 125.86, 123.57, 122.06, 121.17, 53.97, 53.88, 36.79, 36.02, 35.99. (HRMS) (ESI, m/z): calculated for C_25_H_27_N_4_^+^ [M + H]^+^ *m/z=*383.22302; found *m/z=*383.2229.

### Synthesis of [Cu(DQPEA)NCCH_3_](ClO_4_)_2_

To a stirred solution of **[**Cu(OH_2_)_6_](ClO_4_)_2_ (51.0 mg, 0.137 mmol) in MeCN (1.0 mL) was added a solution of DQPEA (50.2 mg, 0.124 mmol) in MeCN (1.5 mL). After standing at room temperature for three days, green crystals suitable for X‐ray analysis were obtained. HRMS (ESI, *m/z*): calculated for C_27_H_24_N_4_Cu^2+^ [M]^2+^ *m/z*=233.5640; found *m/z*=233.5643.

### Synthesis of [Zn(DQPEA)NCCH_3_](ClO_4_)_2_

To a stirred solution of **[**Zn(OH_2_)_6_](ClO_4_)_2_ (50.0 mg, 0.134 mmol) in MeCN (1.0 mL) was added a solution of DQPEA (50.0 mg, 0.123 mmol) in MeCN (1.5 mL). Colourless crystals suitable for X‐ray analysis were obtained on standing at room temperature. ^1^H NMR (400 MHz, CD_3_OD) δ 9.45 (d, *J*=5.4 Hz, 1H), 8.66 (d, *J*=8.5 Hz, 2H), 8.36 (d, *J*=8.7 Hz, 2H), 8.10 (d, *J*=8.2 Hz, 2H), 7.96 (t, *J*=7.9 Hz, 2H), 7.90 (td, *J*=7.8, 1.6 Hz, 1H), 7.73 (dd, *J*=14.5, 7.3 Hz, 5H), 7.28 (d, *J*=7.9 Hz, 1H), 5.15–4.86 (m, 4H), 3.37 (q, *J*=6.8 Hz, 2H), 2.90–2.83 (m, 2H). ^13^C NMR (101 MHz, CD_3_OD) δ 161.77, 150.19, 145.85, 142.99, 142.45, 133.29, 130.41, 130.30, 129.18, 128.69, 126.76, 125.50, 122.04, 63.45, 58.03, 32.91, 0.77. HRMS (ESI, *m/z*): calculated for C_27_H_24_N_4_Zn^2+^ [M]^2+^ *m/z*=234.0646; found *m/z*=234.0641.

### Synthesis of [(Mn(DQPEA))_2_O_2_](ClO_4_)_2_ ⋅ (CH_3_CN)_2_

A solution of DQPEA (56 mg, 0.138 mmol) in MeCN (1.5 mL) was added to a stirred solution of **[**Mn(OH_2_)_6_](ClO_4_)_2_ (50.3 mg, 0.138 mmol) in MeCN (1.0 mL). Standing at room temperature gave dark brown crystals suitable for X‐ray analysis. HRMS (ESI, *m/z*): calculated for C_54_H_48_N_8_O_2_Mn_2_^2+^ [M]^2+^ *m/z*=475.1330; found *m/z*=475.1323.

### Synthesis of [Pd(DQEPEA)Cl]Cl ⋅ 11H_2_O

To a stirred solution of K_2_PdCl_4_ (0.078 g, 0.238 mmol) in H_2_O (1.5 mL) was added a solution of DQEPEA (0.104 g, 0.248 mmol) in CH_3_CN (2 mL) and an instant yellow precipitate was formed. The mixture was heated with stirring at 70 °C for 4 hours and during this time the precipitate dissolved. The yellow solution was left to stand under ambient conditions and after 1 week crystals suitable for X‐ray analysis were obtained. ^1^H NMR (400 MHz, CD_3_OD) δ 9.63 (d, *J=*8.7 Hz, 1H), 9.43 (dd, *J=*5.9, 1.5 Hz, 1H), 8.67 (d, *J=*8.3 Hz, 1H), 8.10 (td, *J=*7.7, 1.6 Hz, 2H), 8.00 (d, *J=*8.4 Hz, 2H), 7.89 (ddd, *J=*8.6, 6.8, 1.6 Hz, 1H), 7.82 (d, *J=*8.2 Hz, 1H), 7.76 (d, *J=*7.8 Hz, 1H), 7.68–7.58 (m, 3H), 7.58–7.52 (m, 1H), 7.49 (d, *J=*7.9 Hz, 2H), 6.86 (d, *J=*8.4 Hz, 1H), 5.09 (ddd, *J=*15.1, 12.9, 6.2 Hz, 1H), 4.22 (ddd, *J=*14.5, 10.7, 4.2 Hz, 1H), 3.87 (ddd, *J=*16.4, 7.7, 5.0 Hz, 2H), 3.77 (ddd, *J=*13.8, 6.4, 2.1 Hz, 1H), 3.56–3.40 (m, 2H), 3.12 (td, *J=*13.4, 3.2 Hz, 1H), 3.05–2.92 (m, 1H), 2.90–2.84 (m, 1H), 2.72 (ddd, *J=*13.1, 10.7, 5.9 Hz, 1H), 2.46 (ddd, *J=*13.0, 11.5, 4.2 Hz, 1H). ^13^C NMR (101 MHz, CD_3_OD) δ 162.43, 160.22, 158.23, 156.06, 148.30, 147.28, 143.85, 142.22, 138.34, 132.83, 130.91, 129.98, 129.80, 129.71, 129.21, 128.88, 128.76, 128.23, 127.57, 126.67, 124.43, 123.39, 122.25, 64.71, 59.84, 57.88, 42.73, 38.72, 37.14. HRMS (ESI, *m/z*): calculated for C_29_H_28_ClN_4_Pd^+^ [M]^+^ *m/z=*573.11129; found *m/z=*573.1115.

### Synthesis of [(DPEA)Co(μ‐OH)_3_Co(DPEA)](ClO_4_)_3_ ⋅ 0.5H_2_O ⋅ MeCN

To a stirred solution of **[**Co(OH_2_)_6_](ClO_4_)_2_ (0.047 g, 0.128 mmol) dissolved in CH_3_CN (1 mL) was added a solution of QEDPEA (0.051 g, 0.133 mmol) dissolved in CH_3_CN (1.5 mL). The resulting solution was stirred at room temperature for 5 minutes and was then left to stand under ambient conditions. After three days crystals suitable for X‐ray analysis were obtained.

## Results and Discussion

### Ligand Syntheses

The DQPEA ligand was prepared by reaction of 2‐(bromomethyl)quinoline with 2‐(pyridine‐2‐yl)ethan‐1‐amine in THF, with subsequent purification by column chromatography giving the ligand as a solid. Characterisation was achieved by ^1^H and ^13^C NMR spectroscopy, and HRMS; these were consistent with the proposed structure. It should be noted that, while two complexes containing the DQPEA ligand have previously been structurally characterised, the ligand synthesis has never been reported.^[26]^

**Figure 2:**
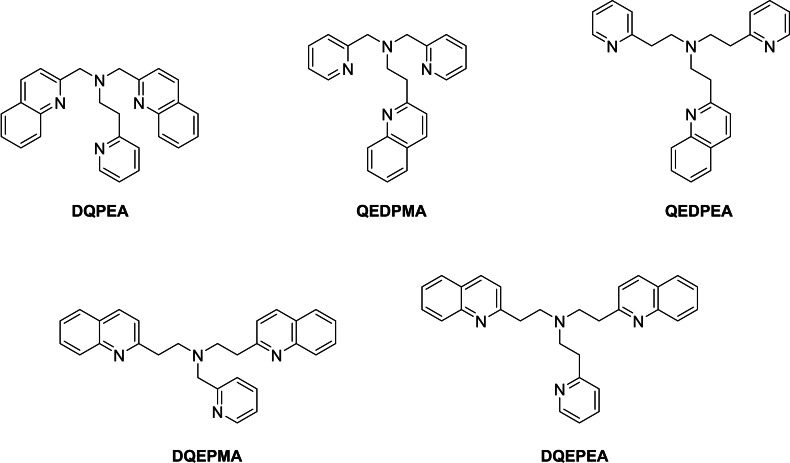
Structures of the quinolyl‐containing ligands.

Initial attempts to make the 2‐quinolylethyl‐containing ligands shown in Figure 2 utilised alkylation with what was reported^[27]^ as free base 2‐(2‐chloroethyl)quinoline to install the 2‐quinolylethyl arms. However, we found the reported compound to in fact be the hydrochloride salt, and all attempts to either convert this directly to the free base, or use this as an alkylating agent in the presence of an *in situ* base gave only formation of 2‐vinylquinoline, presumably formed as a result of a base‐promoted elimination reaction. However, realising the previous use of 2‐vinylpyridine to introduce 2‐pyridylethyl groups into tripodal ligands,[^28^, ^29^, ^30^, ^31^] we successfully used the analogous reaction of 2‐vinylquinoline with the appropriate pyridyl‐containing starting material to prepare the new ligands shown in Figure 2. Chromatography on alumina gave the pure ligand in each case, with characterisation achieved by ^1^H, ^13^C, COSY, HSQC and HMBC NMR, and mass spectrometry.

### Complex Syntheses

**Figure 3:**
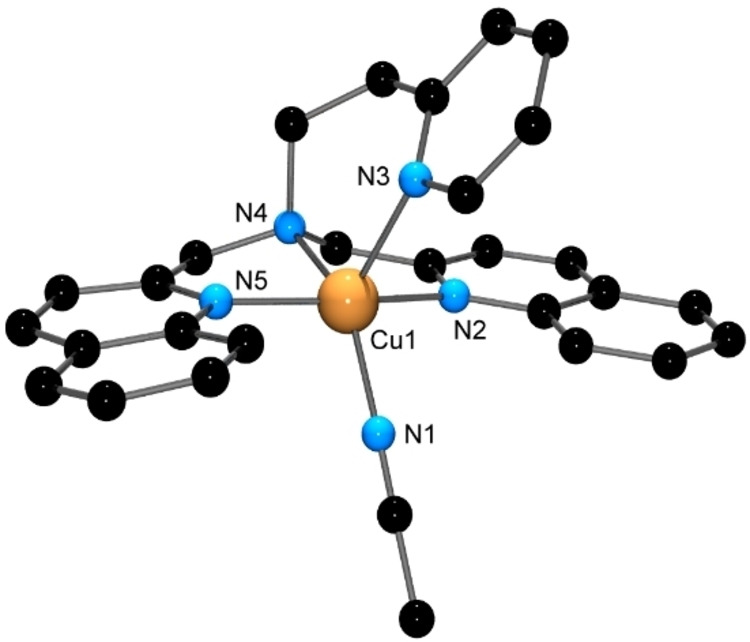
X‐ray structure of the [Cu(DQPEA)NCMe]^2+^ cation. H atoms have been removed for clarity. Selected bond lengths (Å) and angles (°); Cu(1)−N(5) 2.003(4), Cu(1)−N(1) 2.016(3), Cu(1)−N(3) 2.047(3), Cu(1)−N(4) 2.050(3), Cu(1)−N(2) 2.190(3); N(5)−Cu(1)−N(1) 147.52(14), N(5)−Cu(1)−N(3) 93.27(14), N(1)−Cu(1)−N(3) 82.03(13), N(5)−Cu(1)−N(4) 95.77(14), N(1)−Cu(1)−N(4) 82.59(13), N(3)−Cu(1)−N(4) 162.97(13), N(5)−Cu(1)−N(2) 112.46(14), N(1)−Cu(1)−N(2) 99.86(14), N(3)−Cu(1)−N(2) 103.40(13), N(4)−Cu(1)−N(2) 86.42(12).

**Figure 4:**
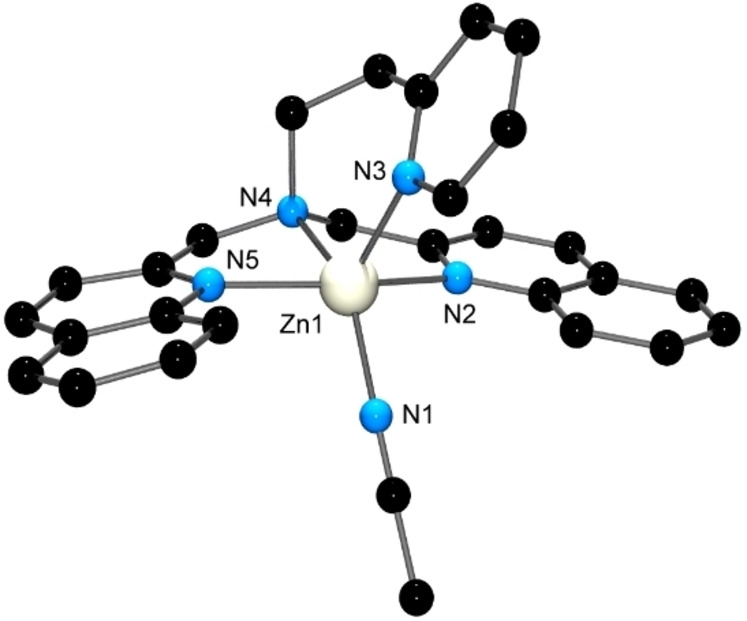
X‐ray structure of the [Zn(DQPEA)NCMe]^2+^ cation. H atoms have been removed for clarity. Selected bond lengths (Å) and angles (°); Zn(1)−N(4) 2.181(3), Zn(1)−N(1) 2.094(3), Zn(1)−N(2) 2.063(2), Zn(1)−N(3) 2.164(3), Zn(1)−N(5) 2.033(3); N(5)−Zn(1)−N(2) 110.17(10), N(5)−Zn(1)−N(1) 146.57(11), N(2)−Zn(1)−N(1) 103.19(10), N(5)−Zn(1)−N(3) 94.48(11), N(2)−Zn(1)−N(3) 104.70(10), N(1)−Zn(1)−N(3) 78.78(11), N(5)−Zn(1)−N(4) 97.84(11), N(2)−Zn(1)−N(4) 89.87(10), N(1)−Zn(1)−N(4) 79.83(10), N(3)−Zn(1)−N(4) 156.36(10).

Reaction of [M(OH_2_)_6_](ClO_4_)_2_ (M=Cu, Zn) with an equimolar amount of the DQPEA ligand in MeCN at room temperature gave X‐ray quality crystals of [M(DQPEA)NCMe](ClO_4_)_2_ on standing, with the structures of the Cu(II) and Zn(II) complexes being similar. Figures 3 and 4 show the structures of the [Cu(DQPEA)NCMe]^2+^ and [Zn(DQPEA)NCMe]^2+^ cations, respectively.

Both cations display a 5‐coordinate geometry, with the metal ion bound to all four nitrogen donor atoms of the DQPEA ligand and the fifth coordination site occupied by a solvent acetonitrile nitrogen atom. This gives a distorted square‐based pyramidal geometry about the metal ion, with τ_5_[^32^, ^33^] values of 0.26 (Cu) and 0.16 (Zn). In both complexes, the metal ion sits above the mean plane defined by the quinoline N atoms, the tertiary aliphatic N atom, and the MeCN N atom, with the Cu ion displaced 0.382 Å towards the pyridine N atom and the Zn ion 0.446 Å. Both complexes display the ‘quinolyl split’^[10]^ in which the N1‐M−N3 angle is bisected by the two quinoline rings; N1 cannot be coplanar with N2, N4 and N5 owing to steric clashes with the C8 protons of the quinoline rings. A similar quinolyl split is observed in the [Zn(DQPEA)(OH_2_)]^2+^ cation.^[26]^

**Figure 5:**
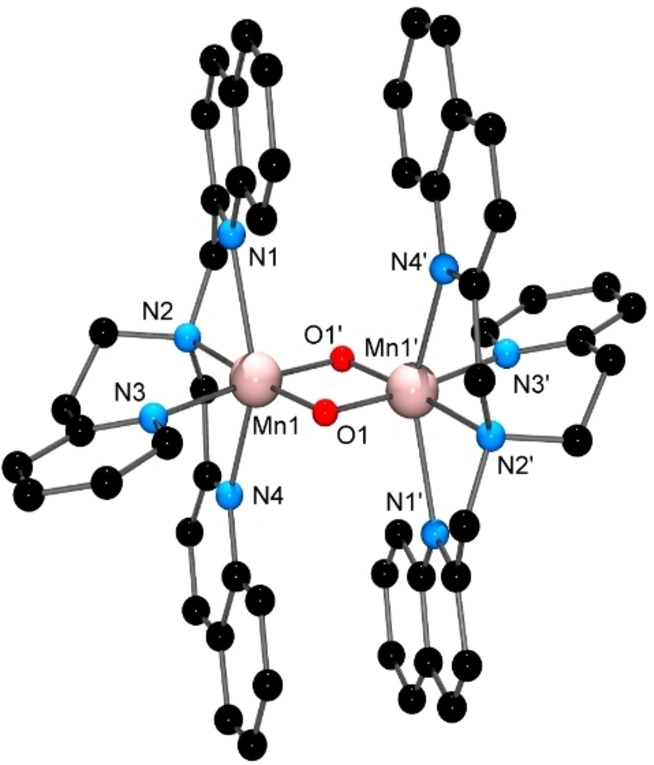
X‐ray structure of one of the two independent [(DQPEA)Mn(μ‐O)_2_Mn(DQPEA)]^2+^ cations in the unit cell of [(DQPEA)Mn(μ‐O)_2_Mn(DQPEA)](ClO_4_)_2_ ⋅ (CH_3_CN)_2_. H atoms have been removed for clarity. Selected bond lengths (Å) and angles (°); Mn(1)−O(1) 1.8267(17), Mn(1)‐O(1’) 1.8343(17), Mn(1)−N(1) 2.333(2), Mn(1)−N(2) 2.144(2), Mn(1)−N(3) 2.147(2), Mn(1)−N(4) 2.312(2), Mn(1)−Mn(1’) 2.6913(7); O(1)−Mn(1)−O(1’) 85.37(8), O(1)−Mn(1)−N(2) 174.15(8), O(1’)−Mn(1)−N(2) 89.21(8), O(1)‐Mn(1)‐N(3) 92.37(8), O(1’)−Mn(1)−N(3) 177.50(8), N(2)−Mn(1)−N(3) 93.09(8), O(1)−Mn(1)−N(4) 105.97(8), O(1’)−Mn(1)−N(4) 93.67(7), N(2)−Mn(1)−N(4) 76.52(8), N(3)−Mn(1)−N(4) 85.91(8), O(1)−Mn(1)−N(1) 103.41(8), O(1’)−Mn(1)−N(1) 92.45(7), N(2)−Mn(1)−N(1) 74.60(8), N(3)−Mn(1)−N(1) 89.11(8), N(4)−Mn(1)−N(1) 150.36(8), Mn(1)−O(1)−Mn(1’) 94.63(8).

The Mn_2_(III,III) complex, [(DQPEA)Mn(μ‐O)_2_Mn(DQPEA)](ClO_4_)_2_ ⋅ (CH_3_CN)_2_, the cation of which is shown in Figure 5, was prepared by air oxidation of a solution containing the DQPEA ligand and [Mn(OH_2_)_6_](ClO_4_)_2_. Air oxidation has also been used to prepare other Mn_2_(III,III) dimers of tetradentate ligands containing quinolyl units.[^34^, ^35^, ^36^] [(DQPEA)Mn(μ‐O)_2_Mn(DQPEA)] (ClO_4_)_2_ ⋅ (CH_3_CN)_2_ has two independent dimeric cations in the asymmetric unit, each comprising two Mn(III) ions bridged by a bis(μ‐oxo) unit with each Mn ion bonded to all four N atoms of a DQPEA ligand to give a distorted octahedral geometry. The fact that the three arms of the DQPEA ligand are not identical leads to isomeric possibilities in 6‐coordinate species, and, in this case, the ligand binds to give the 6‐isomer[^2^, ^37^] at each metal ion, with the unique six‐membered chelate ring lying in the same plane as the μ‐oxo ligands. The quinoline rings from neighbouring ligands exhibit significant parallel displaced π‐π interactions,^[38]^ with centroid‐centroid distances between the aromatic rings ranging from 3.506 Å to 3.749 Å. The Jahn‐Teller effect expected for high‐spin Mn(III) is manifested in the axial Mn−N bond lengths to the quinoline N atoms in both cations; these are at least 0.14 Å longer than those to the pyridine and aliphatic tertiary N atoms in the equatorial plane, and similar axial bond lengthening has been observed in Mn_2_(III,III) complexes of other quinoline‐containing tripodal ligands.[^34^, ^35^] The Mn−Mn distances of 2.6913(7) Å and 2.6946(7) Å are slightly longer than average for Mn_2_(III,III) bis(μ‐oxo) dimers (average=2.674 Å), leading to a concomitant increase in the Mn‐O−Mn angles (94.63(8)° and 94.90(8)°) relative to the average of 93.85°. Interestingly, these distances and angles appear to be diagnostic for the metal oxidation states, with the average Mn−Mn distances for unsupported bis(μ‐oxo) dimers being 2.674 Å, 2.693 Å, and 2.728 Å for Mn_2_(III,III), Mn_2_(III,IV), and Mn_2_(IV,IV) species, respectively, while the corresponding average Mn‐O−Mn angles are 93.85°, 95.80°, and 97.56°. Indeed, the isolation of the Mn_2_(III,III) complex in this case was unexpected, with the majority of complexes containing the bis(μ‐oxo) bridging unit being the mixed valence Mn_2_(III,IV) species, and Mn_2_(IV,IV) being the next most common. It is interesting in this respect to note that all structurally characterised bis(μ‐oxo) dimers containing quinoline donors are either Mn_2_(II,III) or Mn_2_(III,III), suggesting the quinoline unit to be a weaker donor than pyridine.

**Figure 6:**
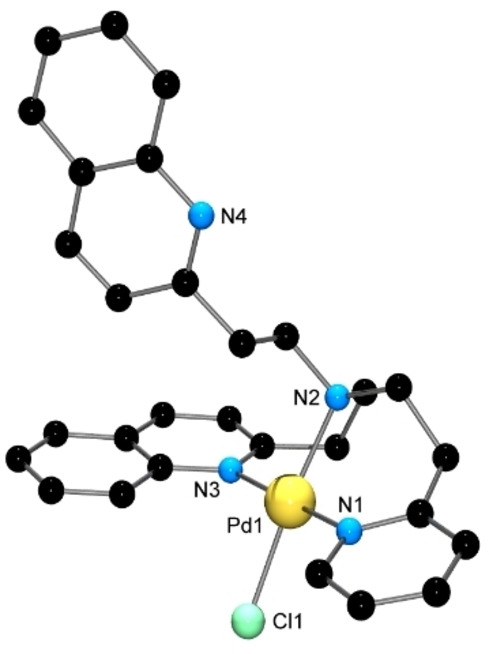
X‐ray structure of one of the two independent [Pd(DQEPEA)Cl]^+^ cations in the unit cell of [Pd(DQEPEA)Cl]Cl ⋅ 11H_2_O. H atoms have been removed for clarity. Selected bond lengths (Å) and angles (°); Pd(1)−Cl(1) 2.3117(9), Pd(1)−N(1) 2.053(3), Pd(1)−N(2) 2.104(3), Pd(1)−N(3) 2.043(3); N(1)−Pd(1)−Cl(1) 89.78(9), N(1)−Pd(1)−N(2) 93.64(12), N(2)−Pd(1)−Cl(1) 170.28(8), N(3)−Pd(1)−Cl(1) 91.25(8), N(3)−Pd(1)−N(1) 176.29(12), N(3)−Pd(1)−N(2) 85.91(11).

It proved difficult to obtain crystalline complexes of complexes of the remaining tripodal ligands, with only the Pd(II) complex shown in Figure 6 amenable to structural characterisation. In the absence of X‐ray structures, both mass spectrometry (Mn^2+^, Co^2+^, Ni^2+^, Cu^2+^, and Zn^2+^) and Job plots (Co^2+^, Ni^2+^, and Cu^2+^) were used to determine the binding stoichiometry of the ligands with the above metal ions; the ligands showed surprisingly low affinity towards Fe^2+^, with brown iron hydroxides precipitating instantly on mixing solutions of ligand and [Fe(OH_2_)_6_](ClO_4_)_2_. The mass spectra of both the free ligands and their *in situ‐*generated metal complexes showed an intense peak at *m*/*z*=156.0 in all cases, consistent with formation of 2‐vinylquinoline via loss of a 2‐quinolylethyl fragment. All complexes observed in the mass spectra had a 1 : 1 metal:ligand stoichiometry, regardless of the metal:ligand mole ratio used, and Job plots were consistent with this in all cases except for reaction of Ni^2+^ with the QEDPMA ligand, where a 1 : 2 metal:ligand ratio was suggested. As none of the ligands contain acidic protons, formation of singly charged species in the mass spectrometer could only be achieved by metal reduction or formation of a ternary [M(L)ClO_4_)]^+^ species. The former was only observed for copper complexes, with [CuL]^+^ species of all ligands being formed. The DQEPEA ligand showed essentially no affinity for Mn^2+^, Co^2+^, Ni^2+^, and Zn^2+^, with the only peaks observed in the mass spectrum of solutions containing these metal ions being those due to the DQEPEA ligand and 2‐vinylquinoline. The amount of the latter was proportionately greater than that observed in the mass spectrum of the free ligand (where in fact 2‐vinylquinoline was the base peak), suggesting that metal‐promoted ligand decomposition was occurring. [ML]^2+^ species were observed for Co^2+^, Ni^2+^ and Zn^2+^ complexes of the QEDPMA ligand, while [M(L)ClO_4_)]^+^ ions were formed from reaction of Mn^2+^, Co^2+^ and Ni^2+^ with the QEDPEA ligand.

The Pd(II) complex [Pd(DQEPEA)Cl]Cl ⋅ 11H_2_O was prepared by heating a mixture of K_2_PdCl_4_ and the ligand in H_2_O/MeCN, and standing the resulting solution at room temperature. The complex was characterized by NMR (^1^H, ^13^C, COSY, HSQC and HMBC), and HR‐ESIMS, and confirmation of the structure was obtained by X‐ray crystallography. A diagram of the cation is given in Figure 6.

The Pd(II) ion is four‐coordinate, and exhibits a slightly distorted square planar geometry (τ_4_=0.09, 0.08),^[39]^ with the DQEPEA ligand adopting a hypodentate[^40^, ^41^] binding mode in which one quinoline ring remains unbound. The bound quinoline ring lies at an angle of ~60° to the mean plane of the donor atoms, presumably as this avoids destablising interactions between H8 of the quinoline moiety and the chlorido ligand, while the analogous angle involving the pyridine ring is ~42°. Both angles are significantly larger than those found in both the [Pd(tpa)Cl]^+^ and [Pd(pmea)Cl]^+^ cations, which are less than 20°.[^42^, ^43^] The quinoline moieties are involved in two types of intercation π interactions; the bound quinolines on adjacent cations undergo parallel displaced π‐stacking, with centroid‐centroid distances ranging from 3.610 Å to 3.785 Å, while the unbound quinolines of neighboring cations similarly π‐stack; owing to disorder in one of these quinolines, the centroid‐centroid distances are less precise, but are of the order of 3.5 Å to 3.7 Å. The Pd−Cl distances of 2.2999(9) Å and 2.3117(9) Å are close to the average for four‐coordinate PdN_3_Cl systems (2.308 Å), while the Pd‐N_aromatic_ lengths (2.037(3) Å, 2.041(3) Å, 2.043 Å(3) and 2.053(3) Å) are slightly longer than average (2.010 Å). The ^13^C NMR of the complex confirms the existence of only a single isomer in solution. The ligand could potentially bind through two quinoline N atoms or one quinoline and one pyridine N atom. Observation of 29 peaks in the ^13^C spectrum confirms the latter as the only isomer present in solution.

**Figure 7:**
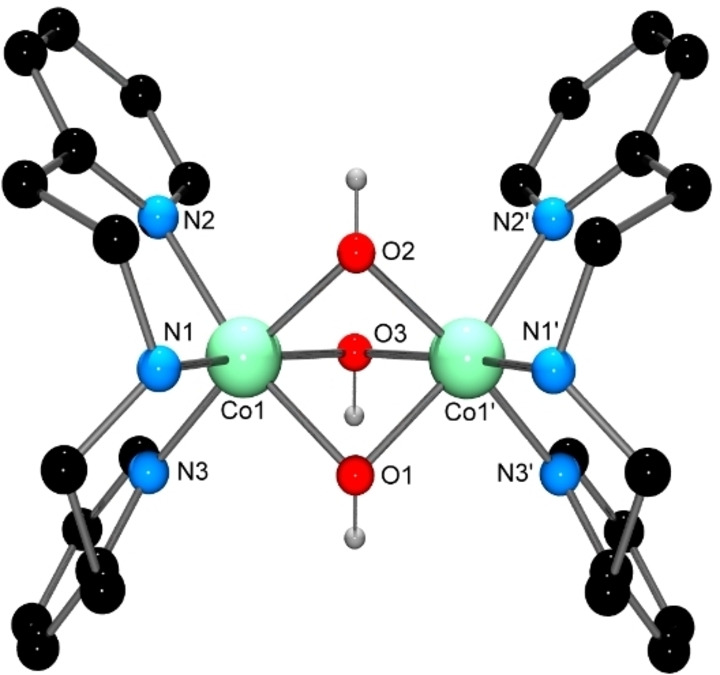
X‐ray structure of the [(DPEA)Co(μ‐OH)_3_Co(DPEA)]^3+^ cation. C−H and N−H hydrogen atoms have been removed for clarity. Selected bond lengths (Å) and angles (°); Co(1)−O(1) 1.9193(15), Co(1)−O(2) 1.8921(15), Co(1)−O(3) 1.9457(15), Co(1)−N(1) 1.9515(18), Co(1)−N(2) 1.9826(18), Co(1)−N(3) 1.9455(18), Co(1)−Co(1’) 2.5946(6); O(1)−Co(1)−O(3) 79.52(7), O(1)−Co(1)−N(1) 87.98(8), O(1)−Co(1)−N(2) 168.63(7), O(1)−Co(1)−N(3) 96.53(7), O(2)−Co(1)−O(1) 77.50(7), O(2)−Co(1)−O(3) 80.83(7), O(2)−Co(1)−N(1) 88.09(8), O(2)−Co(1)−N(2) 91.95(7), O(2)−Co(1)−N(3) 173.07(7), O(3)−Co(1)−N(1) 164.79(7), O(3)−Co(1)−N(2) 94.77(8), N(1)−Co(1)−N(2) 95.99(7), N(3)−Co(1)−O(3) 94.71(7), N(3)−Co(1)−N(1) 95.28(8), N(3)−Co(1)−N(2) 93.72(7), Co(1)−O(1)−Co(1’) 85.05(8), Co(1)−O(2)−Co(1’) 86.57(9), Co(1)−O(3)−Co(1’) 83.64(8).

The Co_2_(III,III) dimer [(DPEA)Co(μ‐OH)_3_Co(DPEA)](ClO_4_)_3_ ⋅ 0.5H_2_O ⋅ MeCN was obtained from the reaction of [Co(OH_2_)_6_](ClO_4_)_2_ with QEDPEA in aerobic conditions, and a diagram of the cation is shown in Figure 7.

Ligand decomposition occurs under the oxidative reaction conditions, resulting in loss of a quinolylethyl moiety and formation of the tridentate DPEA ligand. Metal‐promoted N‐dealkylation of tripodal tetraamine ligands has been reported previously, with both oxidative and hydrolytic routes having been observed.[^44^, ^45^, ^46^] Each 6‐coordinate Co(III) centre displays a slightly distorted octahedral geometry, with the DPEA ligands in an eclipsed configuration relative to each other. The two Co(III) ions are bridged by three hydroxido bridging ligands to give an extremely rare triply‐bridged dimeric core, with a very short Co−Co distance of 2.5946(6) Å. The structure of the complex is well reproduced computationally (Table S2), and investigation of the resulting molecular orbitals shows no obvious evidence of a Co−Co bond. This is in agreement with a previous computational study on a similar tris(alkoxido)‐bridged Co_2_(III,III) complex (*d*_Co…Co_=2.595(6) Å), which likewise gave no evidence for such bonding.^[47]^ The two other examples of complexes containing the tris(hydroxido)‐bridged Co_2_(III,III) core have Co−Co distances of 2.579 Å and 2.549 Å.[^48^, ^49^]

## Conclusions

The new quinoline‐containing tripodal ligands detailed herein are a significant addition to the small number of such ligands known, and the demonstrated efficacy of 2‐vinylquinoline as a synthetic reagent will allow the preparation of new multidentate amine ligands by facilitating the introduction of 2‐quinolylethyl moieties into existing linear and cyclic polyamine ligands.

## Conflict of Interests

The authors declare no conflict of interest.

1

## Supporting information

As a service to our authors and readers, this journal provides supporting information supplied by the authors. Such materials are peer reviewed and may be re‐organized for online delivery, but are not copy‐edited or typeset. Technical support issues arising from supporting information (other than missing files) should be addressed to the authors.

Supporting Information

## Data Availability

The data that support the findings of this study are available from the corresponding author upon reasonable request.
