# Supplementary material for: Structural Variety in Transition Metal Complexes of Tripodal Ligands Containing Mixed Quinolyl and Pyridyl Donors
Source: ChemistryOpen. 2024 Oct 23;14(1):e202400304. doi: 10.1002/open.202400304 (PMC11726704; doi:10.1002/open.202400304)
Supplement: Supplementary file 1 — Supporting Information [file OPEN-14-e202400304-s001.pdf]

ChemistryOpen

Supporting Information

Structural Variety in Transition Metal Complexes of Tripodal Ligands Containing Mixed Quinolyl and Pyridyl Donors

Bronte Carr, Timothy D. Christopher, Tilo Söhnel, Lawrence R. Gahan, Cassandra L. Fleming,* and Allan G. Blackman*

Table S1. Crystal and refinement data for the transition metal complexes.

	$[(DQPEA)Zn(NCMe)](ClO_4)_2$	$[(DQPEA)Cu(NCMe)](ClO_4)_2$	$[(DQPEA)Mn(\mu\text{-}O)_2Mn(DQPEA)](ClO_4)_2.2MeCN$	$\{[(DQEPEA)PdCl]Cl\}_2.12H_2O$	$[(DPEA)Co(\mu\text{-}OH)_3Co(DPEA)](ClO_4)_3.0.5H_2O.MeCN$
Empirical formula	$C_{29}H_{27}Cl_2N_5O_8Zn$	$C_{29}H_{27}Cl_2N_5O_8Zn$	$C_{58}H_{54}Cl_2Mn_2N_{10}O_{10}$	$C_{58}H_{78}Cl_4CoN_8O_{12}Pd_2$	$C_{30}H_{42}Cl_3Co_2N_7O_{15.5}$
Formula weight	709.82	707.99	1231.89	1433.88	972.91
Temperature / K	115(2)	116(2)	116(2)	102(1)	108(1)
Wavelength / Å	1.54184	1.54184	1.54184	1.54184	1.54184
Crystal system	Triclinic	Triclinic	Monoclinic	Monoclinic	Orthorhombic
Space group	P-1	P-1	$P2_1/n$	$P2_1/c$	Pnma
a / Å	8.91680(10)	9.2839(5)	22.0075(3)	14.3699(1) Å	19.3732(3)
b / Å	10.7079(2)	10.6305(4)	12.05850(10)	21.1826(2) Å	11.4254(2)
c / Å	16.9085(2)	16.4412(4)	22.4061(3)	20.6433(2)	17.2501(3)
α / º	97.2950(10)	100.177(2)	90	90	90
β / º	91.8680(10)	91.169(3)	112.5840(10)	92.348(1)	90
γ / º	113.7210(10)	114.056(4)	90	90	90
Volume / $Å^3$	1459.83(4)	1450.65(11)	5490.11(12)	6278.38(10)	3818.25(11)
Z	2	2	4	4	4
Density (calc) / Mg m^{-3}	1.615	1.621	1.490	1.517	1.692
Absorp coeff / mm^{-1}	3.383	3.291	5.224	6.732	9.446
F(000)	728	726	2544	2952	2000
Crystal size / mm^3	0.1 x 0.1 x 0.05	0.08 x 0.08 x 0.05	0.16 x 0.14 x 0.14	0.10 x 0.05 x 0.05	0.08 x 0.04 x 0.04
Theta range	4.564 to 67.716°	4.652 to 67.735º	3.587 to 67.723°	4.174 to 67.732°	3.431 to 72.402°
Index ranges	-10<=h<=10	-11<=h<=11	-26<=h<=26	-12<=h<=17	-12<=h<=23
	-12<=k<=12	-12<=k<=12	-14<=k<=14	-24<=k<=25	-14<=k<=13
	-20<=l<=20	-17<=l<=19	-25<=l<=26	-24<=l<=24	-20<=l<=20
Reflections collected	41034	27171	74196	49780	18769
Independent reflections	5282 [R(int) = 0.0573]	5254 [R(int) = 0.0885]	9948 [R(int) = 0.0661]	11337 [R(int) = 0.0596]	3845 [R(int) = 0.0330]
Completeness to θ = 67.684º / %	99.9	99.9	100.0	99.6	96.9
Absorption correction	Semi-empirical from equivalents	Semi-empirical from equivalents	Semi-empirical from equivalents	Semi-empirical from equivalents	Semi-empirical from equivalents
Max and min transmission	1.00000 and 0.96858	1.00000 and 0.86902	1.00000 and 0.59244	1.00000 and 0.60980	1.00000 and 0.48861
Refinement method	Full-matrix least-squares on F^2	Full-matrix least-squares on F^2	Full-matrix least-squares on F^2	Full-matrix least-squares on F^2	Full-matrix least-squares on F^2
Data/restraints/parameters	5282 / 8 / 417	5254 / 0 / 407	9948 / 14 / 752	11337 / 59 / 674	3845 / 17 / 303
Goodness of fit on F^2	1.065	1.024	1.019	1.052	1.044
Final R indices [I>2sigma(I)]	R1 = 0.0479, wR2 = 0.1265	R1 = 0.0637, wR2 = 0.1703	R1 = 0.0425, wR2 = 0.1050	R1 = 0.0431, wR2 = 0.1144	R1 = 0.0327, wR2 = 0.0852
R indices (all data)	R1 = 0.0544, wR2 = 0.1316	R1 = 0.0725, wR2 = 0.1772	R1 = 0.0484, wR2 = 0.1083	R1 = 0.0489, wR2 = 0.1186	R1 = 0.0372, wR2 = 0.0875
Extinction coefficient	n/a	n/a	n/a	n/a	n/a
Largest diff. peak and hole	1.447 and -0.779 e.$Å^{-3}$	1.424 and -1.249 e.$Å^{-3}$	1.800 and -0.478 e.$Å^{-3}$	1.069 and -1.340 e.$Å^{-3}$	1.039 and -0.653 e.$Å^{-3}$

Table S2

The Cartesian coordinates of the optimized structure for [(DPEA)Co(μ-OH)$_3$Co(DPEA)]$^{3+}$ along with their computed energies. The geometry was computed with M06 functionals using Gaussian 16. The number of imaginary frequencies, the electronic potential energy (E) and the Gibbs free energy at 298.15 K and 1 mol/L (G) are reported. All energies are given in Hartree.

[(DPEA)Co(μ-OH)$_3$Co(DPEA)](ClO$_4$)$_3$
Imaginary frequencies 0

E	-1932.1834072
G 298.15 K, 1 atm	-1931.608742

Co	-1.325752	-0.052630	-0.297514
O	0.000000	-1.374000	-0.828305
O	0.000000	1.008915	-1.206026
O	0.000000	0.128427	1.143712
N	-2.218913	-0.331758	-2.023257
H	-1.364192	-0.507284	-2.554608
N	-2.389220	1.541328	0.238608
N	-2.484472	-1.295877	0.667243
C	-1.977666	2.173185	1.360634
H	-1.109506	1.746817	1.845777
C	-2.608494	3.280228	1.879570
H	-2.229975	3.736824	2.786416
C	-3.722464	3.782346	1.221936
H	-4.252014	4.651060	1.598656
C	-4.135121	3.152910	0.065219
H	-4.992834	3.520237	-0.488352
C	-3.459414	2.035834	-0.417958
C	-3.947952	1.396894	-1.677756
H	-4.536576	2.133783	-2.231366
H	-4.659928	0.600040	-1.423368
C	-2.852389	0.883529	-2.580216
H	-3.262212	0.641465	-3.568571
H	-2.072327	1.637025	-2.718695
C	-3.092549	-1.519892	-2.199035
H	-3.074960	-1.793741	-3.260330
H	-4.120031	-1.233573	-1.960053
C	-2.687016	-2.696995	-1.341600
H	-3.274812	-3.564601	-1.653023
H	-1.639033	-2.956448	-1.524699
C	-2.949378	-2.435918	0.109379
C	-3.703333	-3.329698	0.859133
H	-4.066742	-4.235586	0.385730
C	-3.993903	-3.060657	2.184842
H	-4.586616	-3.754995	2.771213

C	-3.522446	-1.881052	2.742662
H	-3.731793	-1.614603	3.771865
C	-2.775864	-1.031593	1.956042
H	-2.397154	-0.100879	2.361852
H	0.000000	-2.121454	-0.217929
H	-0.000000	1.919868	-0.885401
H	0.000000	-0.655353	1.706624
Co	1.325752	-0.052630	-0.297515
N	2.218913	-0.331758	-2.023257
H	1.364191	-0.507284	-2.554608
N	2.389221	1.541328	0.238608
N	2.484472	-1.295877	0.667243
C	1.977667	2.173184	1.360634
H	1.109506	1.746816	1.845777
C	2.608494	3.280227	1.879570
H	2.229975	3.736823	2.786416
C	3.722465	3.782346	1.221936
H	4.252014	4.651059	1.598656
C	4.135121	3.152910	0.065219
H	4.992834	3.520237	-0.488352
C	3.459414	2.035834	-0.417958
C	3.947952	1.396894	-1.677756
H	4.536575	2.133783	-2.231366
H	4.659928	0.600040	-1.423368
C	2.852389	0.883529	-2.580216
H	3.262211	0.641465	-3.568571
H	2.072327	1.637025	-2.718695
C	3.092549	-1.519892	-2.199035
H	3.074960	-1.793741	-3.260330
H	4.120031	-1.233573	-1.960053
C	2.687016	-2.696995	-1.341600
H	3.274812	-3.564601	-1.653023
H	1.639033	-2.956448	-1.524699
C	2.949378	-2.435918	0.109379
C	3.703333	-3.329698	0.859133
H	4.066742	-4.235586	0.385730
C	3.993903	-3.060657	2.184842
H	4.586616	-3.754994	2.771213
C	3.522446	-1.881052	2.742662
H	3.731793	-1.614603	3.771864
C	2.775864	-1.031593	1.956041
H	2.397154	-0.100879	2.361851

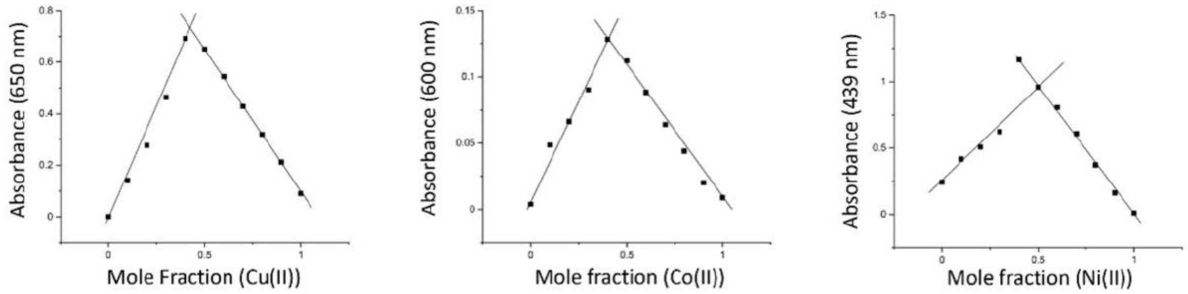

Figure S1: The Job plots for the reaction between the DQEPMA ligand and Cu(II), Co(II) and Ni(II) ions.

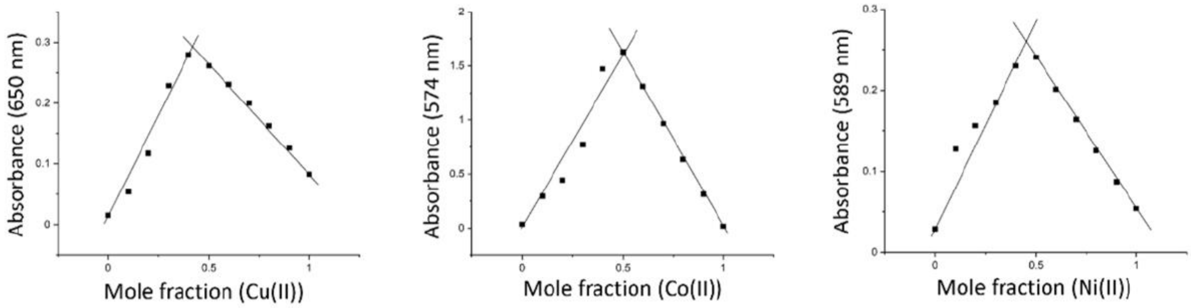

Figure S2: The Job plots for the reaction between the DQEPEA ligand and Cu(II), Co(II) and Ni(II) ions.

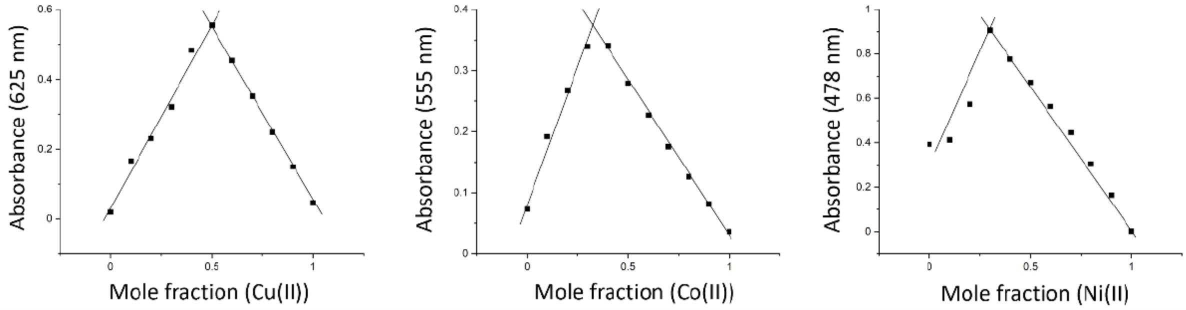

Figure S3: The Job plots for the reaction between the QEDPMA ligand and Cu(II), Co(II) and Ni(II) ions.

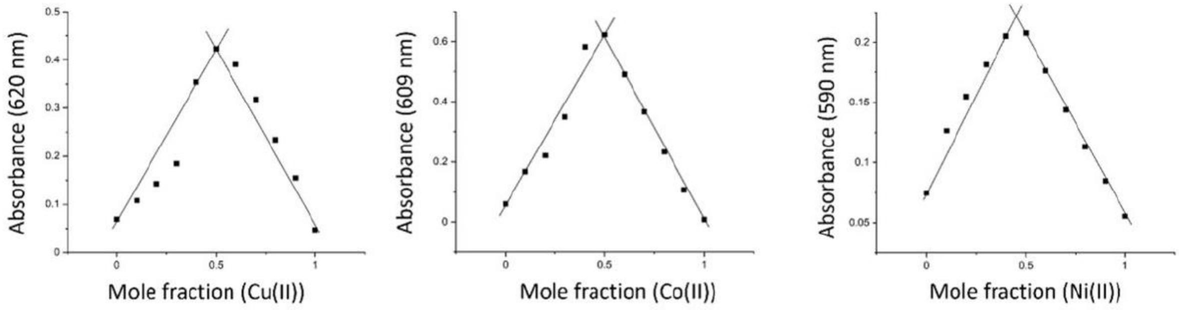

Figure S4: The Job plots for the reaction between the QEDPEA ligand and Cu(II), Co(II) and Ni(II) ions.
